# Survey data on cost and benefits of climate smart agricultural technologies in western Kenya

**DOI:** 10.1016/j.dib.2017.11.027

**Published:** 2017-11-11

**Authors:** S.K. Ng'ang'a, C.M. Mwungu, C. Mwongera, I. Kinyua, A. Notenbaert, E. Girvetz

**Affiliations:** a International Centre for Tropical Agriculture (CIAT), Sub-regional Office for Africa, Pan-Africa Bean Research Alliance National Agricultural, Research Laboratories, Kawanda, P. O. Box 6247, Kampala, Uganda; b International Centre for Tropical Agriculture (CIAT), Regional Office for Africa, Kasarani, Road, ICIPE Complex, P.O. Box 823-00621, Nairobi, Kenya

**Keywords:** Soil, Farm production, Cost and benefit, Climate-Smart soil practices, Kenya

## Abstract

This paper describes data that were collected in three counties of western Kenya, namely Siaya, Bungoma, and Kakamega. The main aim of collecting the data was to assess the climate smartness, profitability and returns of soil protection and rehabilitation measures. The data were collected from 88 households. The households were selected using simple random sampling technique from a primary sampling frame of 180 farm households provided by the ministry of agriculture through the counties agricultural officers. The surveys were administered by trained research assistants using a structured questionnaire that was designed in Census and Survey Processing System (CSPro). Later, the data was exported to STATA version 14.1 for cleaning and management purposes. The data are hosted in an open source dataverse to allow other researchers generate new insights from the data (http://dx.doi.org/10.7910/DVN/K6JQXC).

**Specifications Table**

**Table:** 

Subject area	*Agricultural economics*
More specific subject area	*Soil technologies, Farm production, Cost-benefit, Climate Smart soil technologies*
Type of data	*Tables of household general information and site characteristics, household respondent type, soil practices, trend in physical productivity (business as usual and climate smart soil technology (CSA)), changes in inputs, changes in implementation costs, changes in maintenance cost, changes in operation costs, and change in external effects*
How data was acquired	*Household survey using a structured questionnaire that was designed in CSPro*
Data format	Stata files (containing categorical and numeric variables) in raw format *.dta
Experimental factors	–
Experimental features	–
Data source location	Kenya
Data accessibility	*Data package title: Household Survey Data on Cost Benefit Analysis of Climate-Smart Soil Practices in Western Kenya*
	*Resource link:*https://dataverse.harvard.edu/dataset.xhtml?persistentId=doi:10.7910/DVN/K6JQXC
	*Identifier: doi: 10.7910/DVN/K6JQXC*

**Value of the data**

• The data allows researchers to compute cost benefit indicators of climate-smart soil practices in Western Kenya in order to understand the perceived benefits and cost from a private and social point of view.
• The data can help researchers to understand what explains farmers' decision to implement climate-smart soil (CSS) practices while allowing comparisons with soil management practices from other countries in sub-Sahara Africa.
• This data can provide a significant contribution to the literature on economic assessment of climate-smart agricultural (CSA) technologies on the costs that accrue to the stakeholders in both the short- and long-term.
• The data can be used to identify barriers to adoption and implementation of different soil practices at the micro level and macro level.

## 1 Data

This article presents data collected by CIAT with an aim of assessing the climate smartness, profitability and returns of soil protection and rehabilitation measures. The data was collected using a structured questionnaire by means of Computer Aided Personal Interview (CAPI) technique to make the collection process effective and to minimize errors such as outliers and missing values. It is estimated that researchers spend about 80% of their time cleaning and organizing data [1] and only 20% generating findings from the data. Thus, CAPI technique minimized time spent on transcribing the results into a computer, cleaning and formatting the data for statistical programs that statisticians can use easily. The questionnaire was divided into eight sections. Section 1 provided general information about the study site, section 2 provided demographic data including the household head age, gender, education level, and farming experience, section 3 evaluated farm activities (without intervention), section 4 evaluated implemented CSS practices such as improved seeds, agroforestry, inorganic fertilizers, liming, organic manure, section 5 established yields, prices, inputs and costs of the implementing farming activities (before and after intervention), section 7 described household financial information, and section 8 dealt with the environmental and socio-economic effects.

Data were collected with the help of six local enumerators—two from each of the three counties—who were fluent in both English and the local language. These enumerators trained intensively for five days in English language to ensure a thorough understanding of the research questions and to control loss of information during translation. They were also trained on using CSPro entry android application and how to record geographical positioning coordinates using a GPS. To maintain consistency during the interviews, each enumerator had a hard copy version of the questionnaire to serve as a reference point throughout the survey period and to act as a backup in case of power failure, although data were to be entered directly into the laptops if hard copy questionnaires were used. Each enumerator was given CSPro data entry application on their laptops to facilitate data entry.

Immediately after the training, a reconnaissance was done to test enumerators’ ability in collecting reliable data and time taken to interview one farmer for logistical planning. This served as an important exercise for enumerators to familiarize well with the questionnaire and also for the engaged CIAT staff to have prior knowledge of the field challenges. Enumerators were instructed to interview the household head because they are more conversant with farming activities. In the absence of the household head, however, the enumerators were requested to interview any other household member above 18 years old as long as the member had more than 10 years of farming experience. We defined household head as a person who was the main decision maker as far as farming was concerned and participated in most of the agricultural activities of the household. CSPro was linked to a CIAT staff drop box account that aided data backup and daily checking of the quality of the data. A CIAT research associate in Nairobi office was tasked to check for consistencies in the data using predesigned Stata do files.

## 2 Research design, materials and methods

**Fig. 1:**
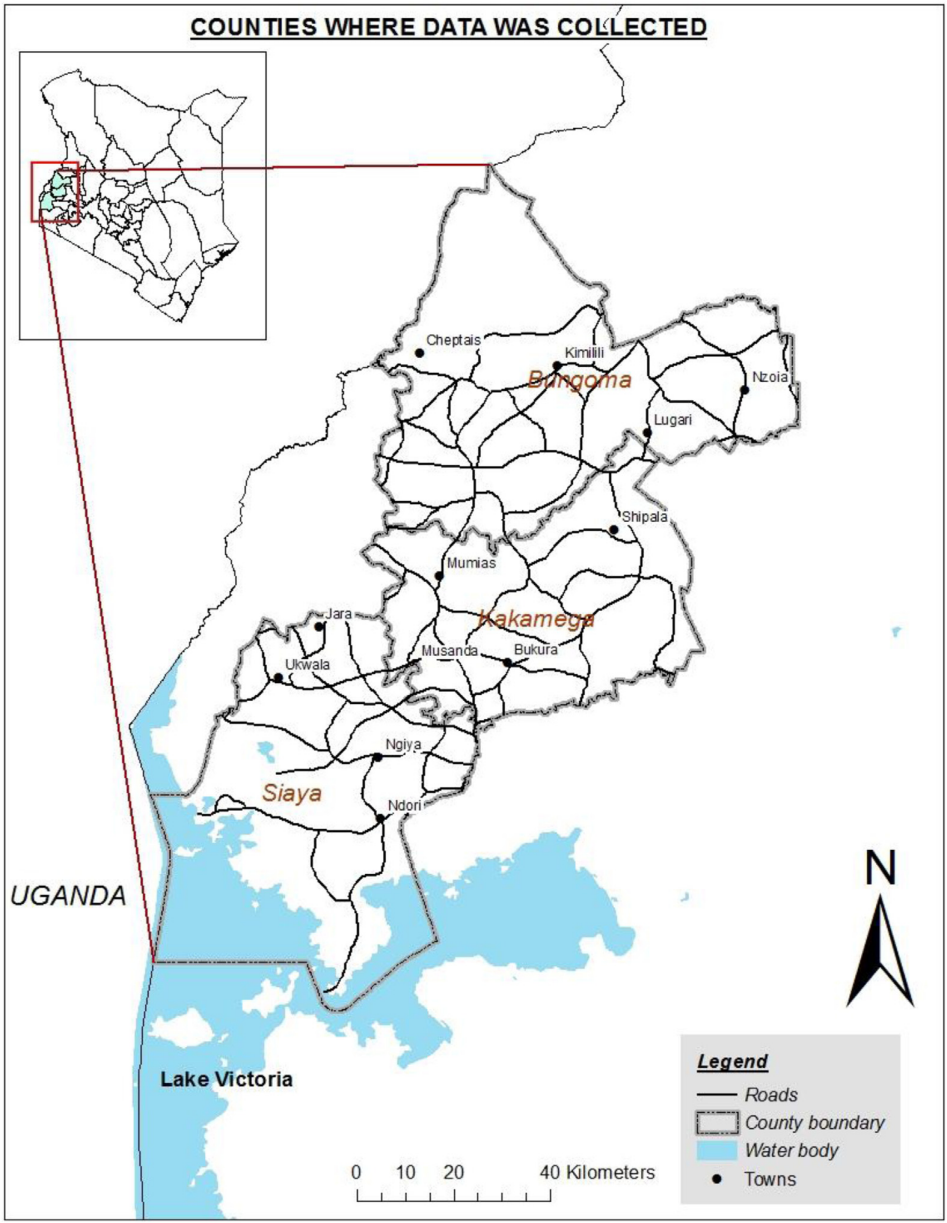
A map of Kenya and the three counties (Siaya, Kakamega, and Bungoma) in western Kenya, where the data was collected.

This data was collected from three counties of western Kenya, namely Bungoma, Kakamega, and Siaya (Fig. 1). Siaya county is located in the former Nyanza province while Bungoma and Kakamega counties are located in the former western province of Kenya. For a detailed description of the study area see [2], [3]. Prior to the data collection, a climate-smart soil practices prioritization workshop was conducted in Kakamega Golf hotel to validate farming typologies in the study area [2] and identify the common practices that are prioritized by both farmers and experts (county agriculture officials, extension officers, NGO representatives in the counties) in each farm typology. Step one involved validation of the main farm typologies. These farm typologies were i) small-scale mixed subsistence farming, ii) medium-scale mixed with commercial dairy, iii) medium-scale mixed with commercial horticulture, iv) medium-scale mixed with commercial cereals, and v) large-scale commercial farming.

Step two involved identification and listing of the already existing (and new) CSS practices that are applicable in the different farm typologies through focus group discussion. Step three involved evaluation of the listed practices using ten indicators in the CSA goals of productivity, resilience, low emission and development. The main goal of step three was to generate a short list comprising three high-interest CSS practices for each farm typology (Table 1). Step four involved identifying common practices prioritized by both the farmers and the experts in each farm typology. Step five involved conducting an in-depth household survey for the selected CSS practices. From steps one to step four resulted a list of 40 CSS practices were gathered, out of which only eight practices were shortlisted – based on the priority ranking based on the 10 indicators of climate smart agriculture prioritization framework (CSA-PF) – for CBA analysis (Table 1). The data in this paper was derived from step ‘five’, by outlining the detailed costs and benefits for short-listed – by farmers and experts for each farm typology – CSS practices This information was supplemented by the Deutsche Gesellschaft für Internationale Zusammenarbeit (GIZ) Kakamega who were closely working with farmers in the region. farmers were randomly selected depending on the farm typology and county of origin using random formulae in Microsoft Excel. Ultimately, a total of 88 households were interviewed as shown in Table 1.

**Table 1:** Farm typologies of the sampled households by County.

Typology	Siaya	Kakamega	Bungoma	Total
Medium-scale mixed commercial dairy	7	8	7	22
Medium-scale commercial horticulture	6	5	5	16
Medium-scale mixed commercial cereals	6	6	5	17
Small-scale mixed subsistence farming	6	8	6	20
Large-scale commercial farming	5	4	4	13
Total	30	31	27	88
