# Supplementary material for: Survey data on cost and benefits of climate smart agricultural technologies in western Kenya
Source: Data Brief. 2017 Nov 11;16:261–5. doi: 10.1016/j.dib.2017.11.027 (PMC5709291; doi:10.1016/j.dib.2017.11.027)
Supplement: Supplementary file 1 — Supplementary material [file mmc1.docx]

**Conflict of interest statement**

**Manuscript title: “Survey data on cost benefit analysis of climate smart soil practices in Western Kenya”**

The authors whose names are listed immediately below certify that they have NO affiliations with or involvement in any organization or entity with any financial interest (such as honoraria; educational grants; participation in speakers’ bureaus; membership, employment, consultancies, stock ownership, or other equity interest; and expert testimony or patent-licensing arrangements), or non-financial interest (such as personal or professional relationships, affiliations, knowledge or beliefs) in the subject matter or materials discussed in this manuscript.

Author names:

Ng'ang'a, Stanley Karanja

Chris Miyonzi Mwungu

Mwongera Caroline

Kinyua Ivy

Notenbaert An

Girvetz, Evan
